# Aortic Arch Reconstruction Using Nonvalved Femoral Vein Homograft in High-Risk Neonates

**DOI:** 10.1177/21501351231176256

**Published:** 2023-09-11

**Authors:** Kyle G. Mitchell, Julija Dobrila, Blaz Podgorsek, Christopher Greenleaf, Peter Chen, Jorge D. Salazar, Damien J. LaPar

**Affiliations:** 1 Division of Pediatric and Congenital Heart Surgery, 12339 University of Texas McGovern Medical School, Houston, TX, USA

## Abstract

Aortic arch obstruction is often present with complex concomitant congenital heart defects (CHDs). The use of nonvalved femoral vein homograft (FVH) to reconstruct the aortic arch has distinct surgical advantages, including simplified reconstruction. We present an intraoperative video of a Yasui procedure utilizing FVH for aortic reconstruction in a 12-day-old (2.2 kg) neonate with right ventricular outflow tract obstruction, malalignment ventricular septal defect, aortic valve atresia, aortic arch hypoplasia, atrial septal defect, and ductal dependent systemic circulation. Further, we report outcomes for a series of three additional neonatal patients with complex CHD and aortic arch obstruction who underwent FVH arch reconstruction.

## Introduction

Surgical repair of neonatal aortic arch obstruction is often complicated by the presence of complex concomitant congenital heart defects. Although traditional Norwood-style arch reconstruction with patch augmentation of the aorta remains a safe and well-established repair,^1^ a more expeditious strategy may be desired in certain high-risk neonates who present with complex defects and anatomic challenges. We describe the use of nonvalved femoral vein homograft (nvFVH) as a conduit for arch reconstruction in a series of four consecutive neonates with arch obstruction and provide an intraoperative video demonstrating technical details of the use of FVH arch reconstruction in a patient undergoing a Yasui procedure.

## Case Series

### Patient 1

**Figure 1.**
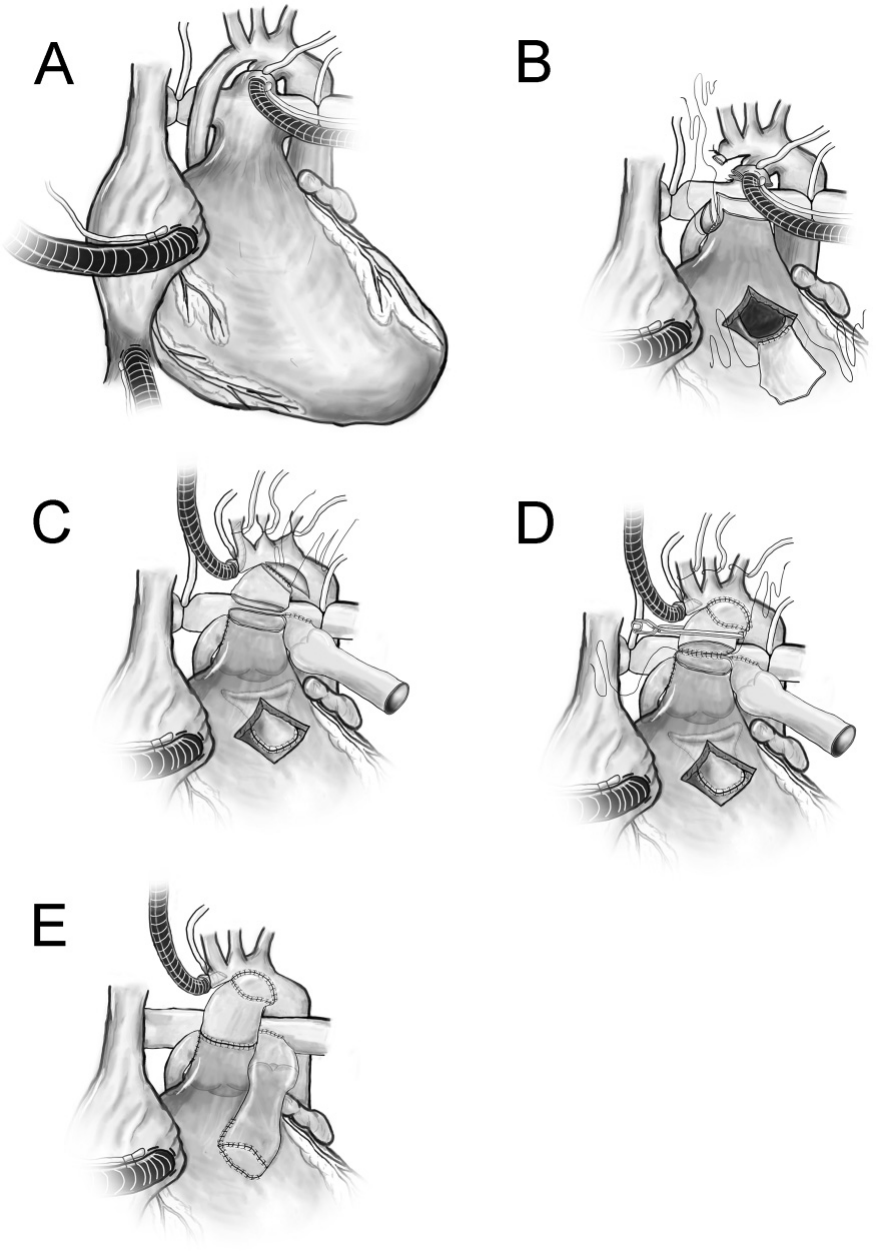
(A) Ductal and bicaval cannulation. (B) Hypoplastic ascending aorta was ligated. A side-to-side Damus-Kaye-Stansel (DKS) anastomosis was fashioned between ascending aorta and main pulmonary artery at right pulmonary sinus. The ventricular septal defect was closed with a pericardial patch and baffled to the DKS. (C) The distal anastomosis using nonvalved femoral vein homograft (FVH) from DKS to lesser curvature of arch (augmented with patch) and distal anastomosis for right ventricle to pulmonary artery conduit (valved FVH). (D) Anastomosis between the nonvalved FVH to the DKS. (E) Demonstration of completed repair. The fenestrated atrial septal defect closure is not shown. DKS, Damus-Kaye Stansel; FVH, femoral vein homograft; RVPAC, right ventricle to pulmonary artery conduit.

**Figure 2.**
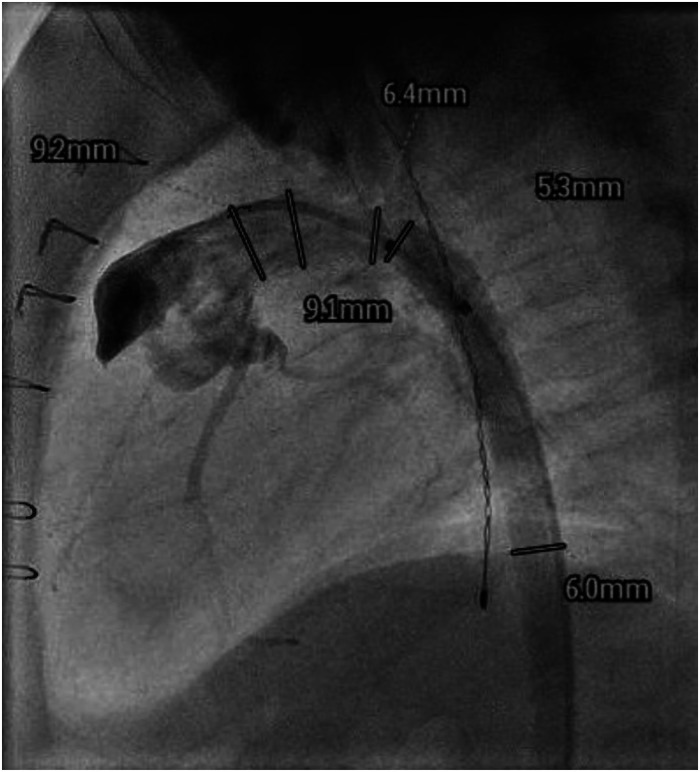
Catheterization (Patient 1, seven months postoperatively) demonstrating unobstructed neoaortic arch.

A 2.2 kg neonate presented with aortic valve atresia, hypoplastic ascending aorta/proximal arch, right ventricular outflow tract (RVOT) obstruction, malalignment ventricular septal defect (VSD), atrial septal defect (ASD), and patent ductus arteriosus (PDA) with ductal-dependent systemic circulation. The patient underwent a Yasui procedure (Figure 1, Video 1) on day of life (DOL) 12, using a 10 mm nvFVH as a conduit to reconstruct the aortic arch, a 10 mm valved FVH (vFVH) as the right ventricle to pulmonary artery conduit (RVPAC), and autologous pericardium to close the VSD and baffle to the Damus-Kaye-Stansel (DKS) anastomosis. Cardiopulmonary bypass (CPB), aortic cross clamp (AXC), and antegrade cerebral perfusion (ACP) times were 293, 186, and 36 minutes, respectively. The patient was extubated on postoperative day (POD) 3 and was discharged home on POD 50. Echocardiogram (seven months postoperatively) demonstrated a patent DKS anastomosis with unobstructed aortic arch, complete closure of the VSD, unobstructed RVOT and RVPAC, bidirectional ASD (3 mm fenestration), and good biventricular function. Catheterization demonstrated unobstructed flow through the neoaortic arch (Figure 2, Video 1).

### Patient 2

A 3.2 kg neonate presented with interrupted aortic arch (IAA) type B, double inlet left ventricle, VSD, aortic valve hypoplasia, functional single ventricle, PDA, and tracheoesophageal fistula. We performed pulmonary artery banding on DOL 4 and definitive repair at DOL 15. The aortic root was augmented with a pericardial patch, a 9 mm nvFVH was used as a conduit from the aortic root to descending aorta, and the distal ascending aorta with arch branches was reimplanted into the neoaortic arch in an end-to-side fashion (CPB, AXC, and ACP times: 125, 82, and 28 minutes, respectively). At eight months follow-up, the patient has remained without evidence of obstruction of the reconstructed arch.

### Patient 3

A 38-day-old, 2.4 kg neonate underwent repair of IAA-type A and truncus arteriosus (TA) with VSD closure and PDA division. nvFVH was used to reconstruct the aorta from the truncal valve to descending aorta, and a vFVH was used as an RVPAC (CPB, AXC, and ACP times: 178, 127, and 35 minutes, respectively). She was discharged home on POD 16. Echocardiogram (18 months postoperatively) demonstrated trace neoaortic valve regurgitation, good biventricular function, and an unobstructed reconstructed aortic arch.

### Patient 4

A 2.5 kg neonate presented with IAA-type B, TA, RVOT obstruction, and PDA. On DOL 40, complete repair of IAA and TA with truncal valve repair was achieved using an 8 mm nvFVH to reconstruct the arch from the neoaortic root to descending aorta. vFVH was used as an RVPAC (CPB, AXC, and ACP times: 452, 338, and 57 minutes, respectively). Echocardiogram (POD 52) demonstrated good ventricular function without aortic arch obstruction. Patient was discharged home on POD 53.

## Comment

Neonates with aortic arch obstruction may present with complex associated cardiac malformations; in some, a means of simplifying and expediting the repair may be desired. To illustrate the technical advantages of the use of FVH as an aortic reconstructive conduit, we present as a representative example a video of a Yasui procedure demonstrating the use of nvFVH as a conduit from the aortopulmonary anastomosis to the aortic arch. The use of nvFVH as a conduit in the Yasui procedure has been described as a means to facilitate expeditious aortic reconstruction.^2^ In select patients, its technical advantages (ease of use, reduced cerebral perfusion or circulatory arrest time, ready availability, and less complex anastomoses) are similarly advantageous in patients requiring concomitant repair of other associated defects (eg, TA [*Patients 3 and 4*]). Although in the case presented in the accompanying video we elected to include a patch at the distal aortic anastomosis and a proximal hood at the RV to PA conduit, the use of FVH as a reconstructive conduit may in some cases obviate the technical need for these steps (by allowing, eg, spatulation of the vein graft at anastomotic sites). In addition, the use of tissue from a single donor may limit antigenic exposure in complex patients for whom a subset may ultimately require transplantation.

The durability of this repair strategy has not been established. In this series, no patient required reintervention on the reconstructed aortic arch at the time of the last follow-up; however, this is limited by the lack of long-term outcome data. On the other hand, the use of FVH as a neoaortic conduit has been described in a large series of patients with hypoplastic left heart syndrome who underwent Norwood reconstruction, with results suggesting an acceptable growth profile and rates of stenosis.^3^ Therefore, it is reasonable to expect that FVH aortic arch reconstruction could be a feasible strategy in highly selected neonates with high-risk arches. However, the ultimate long-term fate of the FVH conduit is unknown, and several possibilities of conduit growth in relation to patient somatic growth exist (FVH stenosis, aneurysmal dilation, proportional growth, and absence of conduit growth with subsequent size mismatch). As such, it must be emphasized that additional long-term data are needed to characterize long-term outcomes and potential size mismatch or dilation of the FVH conduit as patients grow.

In summary, nvFVH may be used successfully as a reconstructive aortic conduit in neonates with aortic arch obstruction who are undergoing complex biventricular repairs. We feel that FVH reconstruction may be most advantageous in the following groups of patients: (1) patients with a history of prematurity; (2) neonates at high risk for neurologic complications who require minimized ACP times; (3) prostaglandin-dependent neonates requiring arch reconstruction in the setting of other high-risk features; (4) those with compromised ventricular function, coronary anomalies, or moderate or more severe atrioventricular valve dysfunction; and (5) those with highly abnormal aortic arch and head vessel anatomy. The use of nvFVH provides an important alternative to conventional arch reconstruction and may permit simplification and expediting of repair in selected patients with complex cardiac malformations.
